# New Insights Into the Response of Metabolome of *Escherichia coli* O157:H7 to Ohmic Heating

**DOI:** 10.3389/fmicb.2018.02936

**Published:** 2018-12-06

**Authors:** Xiaojing Tian, Qianqian Yu, Donghao Yao, Lele Shao, Zhihong Liang, Fei Jia, Xingmin Li, Teng Hui, Ruitong Dai

**Affiliations:** ^1^Beijing Advanced Innovation Center for Food Nutrition and Human Health, College of Food Science and Nutritional Engineering, China Agricultural University, Beijing, China; ^2^Beijing Higher Institution Engineering Research Center of Animal Product, China Agricultural University, Beijing, China

**Keywords:** sublethal injury, untargeted metabolomic analysis, HPLC-MS/MS, lipid metabolism, amino acid metabolism

## Abstract

The objective of this study was to investigate the effects of ohmic heating and water bath heating (WB) on the metabolome of *Escherichia coli* O157:H7 cells at the same inactivation levels. Compared to low voltage long time ohmic heating (5 V/cm, 8.50 min, LVLT) and WB (5.50 min), the high voltage short time ohmic heating (10 V/cm, 1.75 min, HVST) had much shorter heating time. Compared to the samples of control (CT), there were a total of 213 differential metabolites identified, among them, 73, 78, and 62 were presented in HVST, LVLT, and WB samples, revealing a stronger metabolomic response of *E. coli* cells to HVST and LVLT than WB. KEGG enrichment analysis indicated that the significantly enriched pathways were biosynthesis and metabolism of amino acids (alanine, arginine, aspartate, and glutamate, etc.), followed by aminoacyl-tRNA biosynthesis among the three treatments. This is the first metabolomic study of *E. coli* cells in response to ohmic heating and presents an important step toward understanding the mechanism of ohmic heating on microbial inactivation, and can serve as a theoretical basis for better application of ohmic heating in food products.

## Introduction

Thermal treatments, as conventional technology in food processing, are used for the pasteurization, sterilization, dehydration, evaporation, and blanching of foods. Generally, heat energy is generated externally and then transferred into the internal of food by conduction or convection in conventional thermal treatment methods. These methods are time consuming due to slow heat transfer through the product, particularly for larger diameter products, and may lead to overcooked surface and quality deterioration (Jaeger et al., 2016; Kanjanapongkul, 2017). Therefore, there is growing interest in alternative thermal treatment methods, which can avoid these shortcomings (Zell et al., 2010). Ohmic heating in particular is just one of these methods, where the heat is generated directly in the food when electric current passes through conductive food (Hradecky et al., 2017). Compared to conventional thermal treatment methods, the heat of ohmic heating is generated from the internal of the food, therefore it can prevent the surface of the solid food or particles from becoming overheated and preserve sensory attributes of food with a shorter heating time (Kanjanapongkul, 2017).

Besides the superior processing characteristics of ohmic heating, the inactivation effect on microorganisms also attracts many researchers’ interest. Up to now, ohmic heating has been widely applied to inactivate vegetative cells and spores in various food, such as *Salmonella* in buffalo milk (Kumar et al., 2014), *Escherichia coli* O157:H7, *Salmonella* Typhimurium, and *Listeria monocytogenes* in orange and tomato juice (Sagong et al., 2011) and in salsa (Kim and Kang, 2017), *Listeria innocua* in meat (Zell et al., 2010), *Alicyclobacillus acidoterrestris* spores in apple juice (Kim et al., 2017), *Bacillus licheniformis* spores in cloudberry jam (Pereira et al., 2007), and *Bacillus cereus* spores in doenjang (Ryang et al., 2016). Most studies indicated that ohmic heating had a comparable or even better inactivation effect on microorganisms than conventional thermal treatment methods, and the possibility of non-thermal inactivation effect of ohmic heating was also referred (Pereira et al., 2007; Somavat et al., 2012; Tian X.J. et al., 2017). However, few studies have successfully proved the existence of the specific non-thermal effects and the effects of ohmic heating on intracellular content on the molecular level.

In recent years, transcriptomic, proteomic, and metabolomic collectively referred to as functional genomics techniques, are becoming increasingly important in the life sciences. These techniques can give new insights and a better understanding of the biological function of a cell or organism (Wu and Li, 2013). Metabolites (MW < 1000) are the building blocks of DNA, RNA, proteins, and lipids and play important roles in cell metabolism, signaling, and regulation for all organisms (Mousavi et al., 2016), their composition or contents can directly reflect the phenotype changes of living organisms (Tian J. et al., 2017). The metabolomics can generate the metabolic profiles of living systems at a specified time and specific environmental conditions. Recently, microbial metabolomics has received much attention due to its potential applications in a wide range of research areas, and several studies have shown metabolite changes when microorganisms were exposed to limited nutrient (Jozefczuk and Al, 2010; Lu et al., 2016), heat (Jozefczuk and Al, 2010), cold (Jozefczuk and Al, 2010; Alreshidi et al., 2015), bacteriostatic agent (Mousavi et al., 2016), toxic (Planchon et al., 2017), and oxidative stress (Jozefczuk and Al, 2010).

Untargeted metabolomics have the capacity to implicate previously unexplored biochemical pathways in a particular biological condition, because it can simultaneously detect as many metabolites as possible to maximize the opportunity of identifying compounds (Yanes et al., 2011). The purpose of this study was to investigate the metabolomic response of *E. coli* O157:H7 cells exposure to ohmic heating using untargeted metabolomic method. Furthermore, conventional water bath heating was performed in order to compare the metabolomic response of *E. coli* to ohmic heating.

## Materials and Methods

### Bacterial Strain and Culture Conditions

*E. coli* O157:H7 (NCTC 12900) was used as experimental strain. Cell cultures were obtained by the same method as our previous study (Tian et al., 2018b). When cultures reached the mid-exponential growth phase (4 h, OD_600_ = 0.314) they were centrifuged at 5000 × *g*, 4°C for 10 min, washed twice with phosphate buffered saline (pH 7.2 ± 0.1, electrical conductivity 1.23 ± 0.09 S/m, 0.09 M NaCl, 0.03 M Na_2_HPO_4_, and 0.01 M NaH_2_PO_4_, PBS), and then re-suspended in the same PBS as above at final concentration approximately 2 × 10^8^ colony-forming units per milliliter (CFU/mL) before treatment.

### Heat Treatments

#### Water Bath Heating

Water bath heating (WB) was carried out by the same thermostatic water bath equipment, heating cell, and heating method as our previous study (Tian et al., 2018b). In brief, the heating cell was filled with approximately 160 mL *E. coli* cell suspension and was heated at 80°C water bath accompanied by constant shakes (15 times/min) in order to achieve uniform heating. The same inactivation levels among WB and ohmic heating protocols for metabolomic analysis were designed to reduce less than 2 log CFU/mL of *E. coli* cell on tryptone soy agar (TSA, pH 7.3 ± 0.2, HB0177, Qingdao Hope Bio-Technology Co., Ltd). Finally, it required 5.50 min for WB to achieve this inactivation level. After heating, the samples were cooled to 4°C in an ice-water mixture immediately, then the samples were pelleted by centrifugation at 5000 × *g* for 12 min (4°C), quenched using liquid nitrogen immediately, and stored at -80°C until metabolomic analysis. Each treatment was performed in six biological replicates, and samples without any treatment were served as control (CT).

#### Ohmic Heating

The ohmic heating equipment (frequency 50 Hz) used was the same as our previous study (Dai et al., 2013), and the experiment was carried out according to the method of Tian et al. (2018b). Approximately 160 mL *E. coli* cell suspension was assigned to the heating cell and was treated by 10 V/cm and 1.75 min (high voltage short time, HVST), and 5 V/cm and 8.50 min (low voltage long time, LVLT), respectively. After heating, samples were treated for subsequent analysis using the same method as WB samples.

### Measurement of Inactivated and Injured *E. coli* Cells

The inactivation of *E. coli* cells was measured by counting plates according to the methods described in our previous study (Tian et al., 2018b). TSA, thin agar layer (TAL), and selective medium Improved-MacConkey sorbitol agar (IMSA, pH 7.0 ± 0.2, 02–328, BeiJing AoBoXing Bio-Technology Co., Ltd) were used to assess the dead, alive, and sublethal status of *E. coli* cells after different treatments. Samples were diluted serially in sterile 0.85% physiological saline, and an appropriate dilution of 100 μL was spread on the three plates. The plates were incubated at 37°C for 24–48 h before enumeration, and each dilution was performed in duplicate. The sublethal ratio was calculated according to the following equation (1) (Bi et al., 2015):

(1) $$\mathrm{The}\;\mathrm{sublethal}\;\mathrm{ratio}\;(\%)=100-(\mathrm{CFU}/{\mathrm{mL}}_{\mathrm{IMSA}})/(\mathrm{CFU}/{\mathrm{mL}}_{\mathrm{TAL}})\times 100$$

Where CFU/mL_IMSA_ represented the colony counts on the IMSA; CFU/mL_TAL_ represented the colony counts on the TAL.

### Untargeted Metabolomic Analysis

#### Sample Extraction and Preparation

The cell precipitate was added 20 μL L-2-chlorphenylalanine solution (0.3 mg/mL, dissolved in methanol) as internal standard, and then the mixture was transferred to the glass vial with pre-cooled methanol/water (4:1, v/v). The mixture was added 200 μL chloroform and resuspended. The suspension was kept on ice and the cells were disrupted by an ultrasonic cell disruptor (500 W, 6 min, 6 s on, 4 s off) (JY98-IIIN, NingBo XinZhi Bio-Technology Co., Ltd). Then metabolites inside the cells were released by an ultrasonic cleaner for 20 min (TYHD-600, Beijing TianYou HengDa Bio-Technology Co., Ltd). After ultrasound, the suspension was centrifuged at 10,000 × *g*, 4°C for 15 min, and the supernatant was collected. One mL of supernatant was added into 1.5 mL centrifuge tube (twice, 0.5 mL each time) and was volatilized. Dried extracts were dissolved in 250 μL methanol/water (7:3, v/v) followed by vortex 30 s, ultrasonic 2 min. Next, the suspension was centrifuged at 10,000 × *g*, 4°C for 15 min, and 180 μL of supernatant was transferred to the sample bottle with lining tube for HPLC-MS/MS analysis.

#### HPLC-MS/MS Analysis

Metabolites of *E. coli* cells with different treatments were analyzed by instrument platform of ultra-high-performance liquid chromatograph-dual pressure linear well-electrostatic field orbital well tandem mass spectrometer (UHPLC-LTQ Orbitrap, Thermo Fisher Scientific, United States). An acquity BEH C18 column (100 mm × 2.1 mm, i.d., 1.7 μm; Waters, Milford, CT, United States) was used. The mixed mobile phase contained formic acid-aqueous solution (0.1%, v/v, A) and acetonitrile solution containing 0.1% formic acid (v/v, B), and gradient elution was as follows: 5–25% B over 0–1.5 min, 25–100% B over 1.5–10 min, holding at 100% B over 10–13 min, 100–5% B over 13–13.5 min, and holding at 5% B over 13.5–14.5 min. The column was maintained at 45°C. Injection volume was 3.00 μL and flow rate was 0.40 mL/min.

An electrospray ionization (ESI) source in either positive or negative ion mode was used to acquire mass spectra profiles. The electrospray capillary voltage, input voltage, and collision voltage were 3.0 kV, 40 V, and 30 eV, respectively. The capillary and ion source temperature were all set at 350°C, with a carrier gas flow rate of 45 L/h. The acquired mass data was collected from 50 to 1000 m/z with the resolution of 30,000.

Quality control (QC) sample was prepared by mixing all samples equivalently to be a pooled sample, and then analyzed using the same method with the analytic samples. The QC samples were injected at regular intervals (every 10 samples) throughout the analytical run to provide a set of data from which repeatability could be assessed.

#### Data Processing

The metabolomics processing software progenesis QI (Waters Corporation,Milford, CT, United States) was used for baseline filtering, peak identification, integration, retention time correction, peak alignment, and normalization of the data sets. The retention time, mass ratio, and peak intensity was obtained.

The positive and negative data were combined to get a combine data set which was imported into SIMCA-P+ 14.0 software package (Umetrics, Umeå, Sweden). Firstly, the unsupervised principle component analysis (PCA) was carried out to visualize the overall distribution of the samples and the stability of the whole analysis process. Then, the supervised (orthogonal) partial least-squares-discriminant analysis [(O) PLS-DA] was performed to find the inter-group differential metabolites. Variable importance in projection (VIP) ranked the overall contribution of each variable to the (O) PLS-DA model, and those variables with VIP > 1 were considered relevant for group discrimination. In this study, the default 7-round cross-validation was applied with 1/seventh of the samples being excluded from the mathematical model in each round, in order to guard against overfitting. R^2^ and Q^2^ values were used to evaluate the accuracy and predictive ability of the models.

Multidimensional analysis of (O) PLS-DA and single-dimensional analysis (student *t*-test) were used to screen the inter-group differential metabolites (VIP > 1, *P* < 0.05). The metabolites were identified using human metabolome database^1^ and METLIN database^2^. Then differential metabolites were annotated by KEGG database, including participating pathway and functional classification.

### Statistical Analysis

One-way variance (ANOVA) analysis was carried out by SPSS 21 software (IBM, United States), and results were considered to be statistically significant at *P* < 0.05. All experiments were performed in sextuplicate, and the values were given as means ± standard deviation of six replicates.

## Results and Discussion

### Inactivation and Sublethal Injury of *E. coli* Cells

In order to obtain appropriate treatment conditions for the metabolomic analysis, *E. coli* cells were HVST, LVLT and WB-treated for 1.75, 8.50, and 5.50 min with final temperature of 57.90, 58.60, and 59.50°C, respectively (Table 1). Under these treatment conditions, cell counts ranged from 6.81 to 6.83 log CFU/mL on TSA, ranged from 6.40 to 6.48 log CFU/mL on TAL, and ranged from 5.57 to 5.60 log CFU/mL on IMSA. There was no significant difference of logarithmic reduction on the same plate among HVST, LVLT, and WB-treated samples with similar final temperature (*P* < 0.05). The initial *E. coli* populations were 8.24, 8.16, and 8.07 log CFU/mL on TSA, TAL, and IMSA, respectively. There should be no significant difference of colony counts on TSA, TAL, and IMSA if no sublethally injured cell was induced by the three treatments (Chueca et al., 2015; Tian et al., 2018a). However, as shown in Table 1, the counts of *E. coli* cells on TSA and TAL were higher than that of IMSA after HVST, LVLT, and WB treatments, which indicated the existence of sublethal injury cells after the three treatments. Based on the difference of plate counts on TSA before and after treatments, there were more than 90% of the *E. coli* cells killed. According to Equation (1), more than 80% cells was sublethally injured after the three treatments (Table 1). At this inactivation degree, there were enough living cells and a large proportion of sublethally injured cells could respond to heat treatments. Because these treatments provided appropriate conditions for metabolomic analysis of *E. coli* cells.

**Table 1:** Inactivation of *E. coli* O157:H7 cells by HVST, LVLT, and WB treatments.

Treatments	Time (min)	Temperature (°C)	Plate counts (log CFU/mL)			Sublethal ratio (%)
				
			TSA	TAL	IMSA	
CT			8.24 ± 0.13^b^	8.16 ± 0.08^b^	8.07 ± 0.11^b^	
HVST	1.75	57.90 ± 0.20^a^	6.81 ± 0.07^a^	6.46 ± 0.06^a^	5.57 ± 0.07^a^	84.11 ± 0.50^a^
LVLT	8.50	58.60 ± 0.51^ab^	6.83 ± 0.07^a^	6.40 ± 0.14^a^	5.60 ± 0.04^a^	80.50 ± 3.10^a^
WB	5.50	59.50 ± 0.70^b^	6.81 ± 0.06^a^	6.48 ± 0.06^a^	5.58 ± 0.03^a^	83.07 ± 1.87^a^


*Values were means ± standard deviation of six replicates. Values in the same column with different letters (a-b) were significantly different (P < 0.05).*

In this study, the heating time for HVST was shorter than that of LVLT with the same inactivation levels of *E. coli* cells, which suggested that a higher voltage gradient could cause a comparable inactivation effect of *E. coli* cells at the similar final temperature with a shorter heating time. Our results were similar to those reported by other researchers. Kim et al. (2018) reported that increasing treatment voltage gradients (9.43–12.14 V_rms_/cm) was an effective way to inactivate *E. coli* O157:H7, *S*. Typhimurium, and *L. monocytogenes* by continuous-type pulsed ohmic heating in buffered peptone water and tomato juice at final temperature of 80°C. When 30, 40, and 50 V/cm voltage gradients were used to inactivate *Alicyclobacillus acidoterrestris* spores in orange juice, Baysal and Icıer (2010) found that the higher voltage gradient had a more effective inactivation effect on *A. acidoterrestris* spores at final temperature of 70°C. Lee et al. (2012) also suggested that the most effective treatment voltage was 40 V/cm compared to 30 and 35 V/cm for inactivating *E. coli, S.* Typhimurium, and *L. monocytogenes* in orange juice and tomato juice at final temperature of 75.5°C.

### Multivariate Statistical Analysis

**FIGURE 1:**
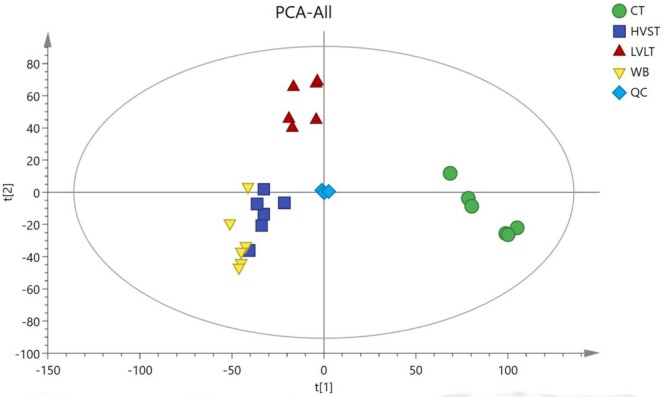
Principal component analysis (PCA) of metabolites from CT, HVST, LVLT, and WB-treated *E. coli* O157:H7 cells. t[1] was the first principal component, and t[2] was the second principal component.

**FIGURE 2:**
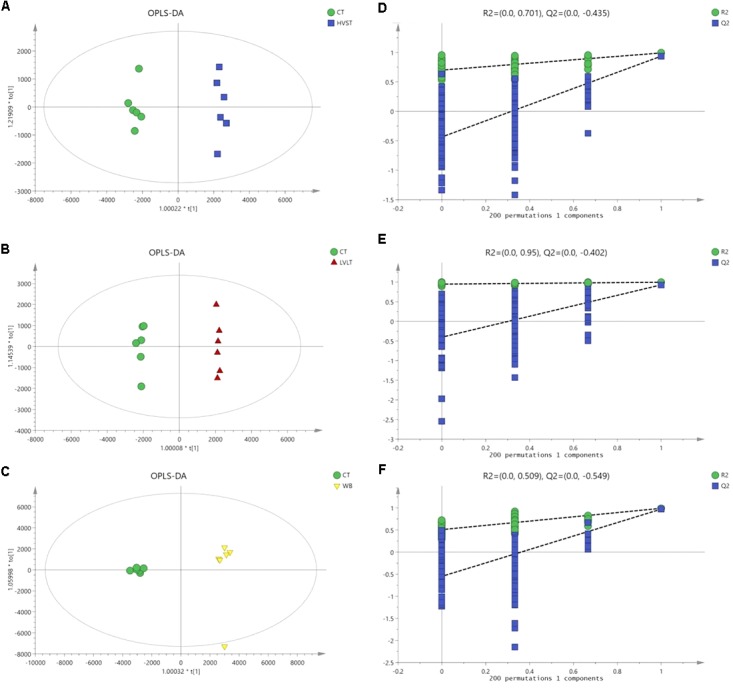
OPLS-DA modeling of metabolites from *E. coli* O157:H7 cells. S-plots **(A–C)** showed the analysis of datasets for HVST vs. CT, LVLT vs. CT, and WB vs. CT treated samples, respectively. t[1] was the first principal component (predicted principal component), and to[1] was the second principal component (orthogonal principal component). **(D–F)** Permutation testing confirmed the good quality of each model.

Metabolome-based class separation was presented in the PCA score plot (Figure 1), the HVST, LVLT, and WB-treated samples were separated from the CT samples. (O) PLS-DA (Figure 2) models were developed for comparison of HVST vs. CT, LVLT vs. CT, and WB vs. CT treated samples. The models displayed good descriptive and predictive abilities, expressed as follows: R^2^(Y) = 0.992, and Q^2^= 0.937 in HVST vs. CT; R^2^(Y) = 0.997, and Q^2^ = 0.931 in LVLT vs. CT; R^2^(Y) = 0.991, and Q^2^ = 0.973 in WB vs. CT (data was not shown).

Statistical analysis indicated that after the three treatments, a total of 213 metabolites demonstrated the existence of metabolomic differences with VIP > 1 and *P* < 0.05, as compared with CT samples (Table 2). Of the 213 differentially expressed metabolites, 73, 78, and 62 metabolites belonged to HVST vs. CT, LVLT vs. CT, and WB vs. CT treated samples, respectively. These metabolites were distributed into 6 functional groups: lipid metabolism (20 metabolites), amino acid metabolism (30 metabolites), nucleotide metabolism (32 metabolites), energy metabolism (8 metabolites), carbohydrate metabolism (8 metabolites), and others (8 metabolites), and some metabolites might belong to more than one category. Moreover, there were 46 overlapping compounds among HVST, LVLT, and WB treatments.

**Table 2:** List of differential metabolites from HVST vs. CT, LVLT vs. CT, and WB vs. CT treated *E. coli* O157:H7 cells.

Metabolites	Retention time (min)	m/z	Ion mode	HVST vs. CT		LVLT vs. CT		WB vs. CT	
						
				VIP	Trend	VIP	Trend	VIP	Trend
**Lipid Metabolism**									
Arachidic acid	8.79	335.2928	POS	1.72	↑	2.60	↑		
Arachidonic acid	6.85	339.1996	NEG			2.29	↑	3.55	↑
CPA(18:0/0:0)	9.55	421.2694	POS			1.21	↑		
D-Glycerate 3-phosphate	0.76	184.9859	NEG	1.43	↑	2.08	↑		
LysoPC(14:1(9Z))	6.99	466.2905	POS	11.61	↑			12.89	↑
LysoPC(14:1(9Z))	6.85	464.2778	NEG	6.81	↑			5.07	↑
LysoPC(15:0)	8.48	482.3221	POS			2.93	↑	3.47	?
LysoPC(15:0)	8.23	480.3090	NEG	1.73	↑	1.56	↑	1.45	↑
LysoPE(0:0/14:0)	6.16	426.2595	POS	7.90	↑			8.31	↑
LysoPE(0:0/14:0)	6.16	424.2466	NEG	4.46	↑			3.30	↑
LysoPE(0:0/14:1(9Z))	5.77	422.2309	NEG	1.13	↑				
LysoPE(0:0/14:1(9Z))	5.66	424.2439	POS	2.02	↑	1.27	↓	2.58	↑
LysoPE(0:0/15:0)	6.63	440.2751	POS	8.39	↑	3.51	↑	8.45	↑
LysoPE(0:0/15:0)	6.58	438.2622	NEG	4.37	↑	1.69	↑	3.25	↑
LysoPE(0:0/16:0)	6.94	454.2907	POS	6.97	↑	4.49	↑	6.57	↑
LysoPE(0:0/16:0)	7.05	452.2779	NEG	10.95	↑			7.62	↑
Myristic acid	11.72	227.2017	NEG					3.07	↑
PE(14:0/18:3(6Z,9Z,12Z))	12.78	684.4601	NEG			1.36	↑		
PE(14:1(9Z)/15:0)	13.06	646.4447	NEG			2.48	↑		
Sphinganine	6.94	302.3036	POS	1.14	↓			10.56	↑
**Amino Acid Metabolism**									
Argininosuccinic acid	3.23	291.1439	POS	2.34	↓				
D-Ala-D-Ala	0.66	161.0914	POS	5.22	↓	4.11	↓	4.40	↓
D-Ala-D-Ala	0.85	159.0778	NEG	3.50	↓	3.08	↓	2.90	↓
Glutathione	0.73	306.0766	NEG	1.24	↓	1.17	↓	1.11	↓
Indole	2.47	118.0646	POS			2.83	↑		
Ketoleucine	2.08	129.0559	NEG			1.18	↑		
L-Arginine	0.94	173.1047	NEG	1.13	↓	1.07	↓		
L-Arginine	0.59	175.1183	POS	1.60	↓	1.41	↓	1.30	↓
L-Asparagine	2.47	155.0444	POS			1.02	↑		
L-Glutamate	0.66	148.0598	POS	4.64	↓	4.43	↓	3.84	↓
L-Glutamate	1.21	130.0876	NEG	2.07	↓	2.25	↓	2.14	↓
L-Glutamine	0.63	169.0586	POS	1.02	↓	1.12	↓		
L-Histidine	0.58	156.0761	POS	1.07	↓				
L-Isoleucine	1.18	132.1014	POS	9.47	↓	10.12	↓	7.69	↓
L-Isoleucine	1.71	164.0719	NEG	2.74	↓	2.88	↓	1.68	↓
L-Lysine	0.60	169.0941	POS					1.25	↓
L-Lysine	0.94	145.0986	NEG	1.14	↓	1.03	↓		
L-Methionine	0.69	150.0576	POS	2.78	↓	2.62	↓	2.28	↓
L-Methionine	0.80	146.0462	NEG	2.58	↓	2.54	↓		
L-Methionine	0.93	148.0441	NEG					1.31	↓
L-Phenylalanine	1.61	166.0856	POS	10.42	↓	10.33	↓	8.54	↓
L-Phenylalanine	0.93	148.0441	NEG	1.51	↓	1.53	↓	2.25	↓
L-Proline	0.66	116.0701	POS	2.68	↓			2.17	↓
L-Tryptophan	2.38	205.0963	POS	1.89	↓	1.73	↓	1.56	↓
L-Tyrosine	0.90	182.0805	POS	2.72	↓	2.51	↓	2.22	↓
L-Valine	0.90	116.0721	NEG	1.15	↓	1.22	↓		
L-Valine	0.86	118.0858	POS	3.14	↓	3.21	↓	2.54	↓
Ornithine	1.12	155.0809	POS	1.71	↓	2.13	↓	1.25	↓
β-Alanine	0.66	90.0544	POS	2.15	↓			1.82	↓
γ-Aminobutryic acid	0.63	126.0521	POS			1.28	↓	1.49	↓
**Nucleotide Metabolism**									
Adenine	0.66	136.0612	POS	8.59	↑	11.40	↑	7.72	↑
Adenine	1.22	170.0241	NEG	1.20	↑	1.40	↑		
Adenosine	0.86	268.1030	POS	1.10	↓			1.11	↓
Adenosine	1.71	302.0661	NEG			1.04	↑		
Adenosine	0.73	505.9882	NEG			2.96	↑		
cAMP	1.22	328.0450	NEG	2.06	↑	1.76	↓	1.47	↑
ADP	0.77	426.0221	NEG			4.21	↑		
ADP	0.89	428.0355	POS			1.23	↑		
ATP	1.22	328.0450	NEG			2.33	?		
Cytosine	0.66	112.0501	POS			1.37	↑		
5′-CMP	0.77	322.0447	NEG			1.14	↑		
CTP	0.73	481.9766	NEG			1.04	↑		
Guanine	0.86	152.0560	POS	1.14	↑				
cGMP	0.86	346.0533	POS	1.06	↑	1.19	↑	1.06	↑
cGMP	0.89	344.0401	NEG	1.19	↑	1.41	↑		
dGMP	0.84	348.0691	POS	1.07	↑	2.19	↑	1.15	↑
dGMP	0.85	346.0557	NEG	1.37	↑			1.18	↑
dGMP	0.86	268.1030	POS			1.74	↑		
Hypoxanthine	0.86	137.0452	POS	5.93	↓			5.87	↓
Hypoxanthine	0.90	135.0316	NEG	1.61	↓	1.09	↓	1.48	↓
Inosine	0.90	291.0688	POS					1.34	↓
Inosine	0.79	323.0287	NEG			1.82	↑	1.53	↓
Uracil	0.67	113.0340	POS	1.79	↓	1.98	↑	1.68	↓
Uracil	0.91	111.0203	NEG	4.19	↓			3.84	↓
UMP	2.48	784.1488	NEG			2.85	↓		
UDP	0.76	402.9948	NEG			2.40	↑		
Thymine	1.03	127.0497	POS	1.27	↓	1.28	↓	1.04	↓
Flavin adenine dinucleotide	2.48	784.1488	NEG	2.90	↓	3.07	↑	2.40	↓
Flavine mononucleotide	2.69	457.1104	POS			1.12	↓	1.49	↓
Flavine mononucleotide	2.68	455.0969	NEG					1.16	↓
Xanthine	1.17	151.0264	NEG	1.62	↓			1.42	↓
Xanthosine	2.71	285.0970	POS	1.05	↓	1.25	↓	1.10	↓
**Carbohydrate Metabolism**									
Citric acid	0.73	191.0200	NEG	1.33	↑	2.48	↑		
Malic acid	0.80	133.0146	NEG	2.13	↓	1.92	↑	1.97	↓
Succinic acid	0.77	117.0197	NEG	7.50	↓	8.24	↓	6.08	↓
Lactic acid	13.95	124.9996	NEG					1.24	↓
D-Ribulose	0.63	173.0413	POS	1.39	↑	1.04	↑		
L-Rhamnulose	0.62	187.0621	POS	1.18	↓	1.00	↓		
UDP-glucose	0.76	565.0477	NEG	4.46	↓	4.42	↓	4.37	↓
α-ketoisovaleric acid	2.47	117.0568	POS			1.25	↑		
**Energy Metabolism**									
cAMP	1.22	328.0450	NEG	2.06	↑	1.76	↓	1.47	↑
ADP	0.77	426.0221	NEG			4.21	↑		
ADP	0.89	428.0355	POS			1.23	↑		
ATP	1.22	328.0450	NEG			2.33	?		
Inosine	0.90	291.0688	POS	1.51	↓	1.08	↓		
Flavin adenine dinucleotide	2.48	784.1488	NEG	2.90	↓	3.07	↑	2.40	↓
Flavine mononucleotide	2.69	457.1104	POS			1.12	↓	1.49	↓
Flavine mononucleotide	2.68	455.0969	NEG					1.16	↓
**Others**									
Niacin	0.70	124.0388	POS	1.80	↓	2.04	↓	1.44	↓
Niacinamide	0.66	123.0548	POS	6.63	↓	5.88	↓	6.21	↓
Pantothenic acid	1.71	220.1171	POS	5.94	↓	6.59	↓		
Pantothenic acid	1.17	218.1036	NEG	1.90	↓	2.12	↓		


### Classification Analysis of Differential Metabolites

#### Lipid Metabolism

Lipid-based metabolism is vital to many biochemistry reactions and related to many biological functions, especially essential to the formation of cell membrane. There were 14, 12, and 14 lipid-metabolism-related differential compounds after HVST, LVLT, and WB treatments, respectively, and most of them were up-regulated, expect for sphinganine and lysoPE (0:0/14:1(9Z)) (POS mode). Among them, only arachidic acid, cPA (18:0/0:0), D-Glycerate 3-phosphate, and myristic acid belong to saturated fatty acids, all the others belong to unsaturated fatty acids. Lysophosphatidylcholine (lysoPC), derived from the hydrolysis of phosphatidylcholines (PC) by phospholipase A2, is of great importance to the cell and participates in many physiological functions (Liu et al., 2013). An increase of lysoPC, such as lysoPC [14:1(9Z)] and lysoPC (15:0) indicated a disturbance of phospholipid catabolism in *E. coli* cells. Lysophosphatidylethanolamine (lysoPE), a constituent of cell membranes, derived from the hydrolysis of phosphatidylethanolamines (PE), which is catalyzed by phospholipase A2 (Tepperman and Soper, 1999). The up-regulation of lysoPE (0:0/14:0), lysoPE [0:0/14:1(9Z)], lysoPE (0:0/15:0), and lysoPE (0:0/16:0) indicated the changes of phospholipid metabolism or cellular damage. Sphinganine involved in the pathway of sphingolipid metabolism, was up-regulated after WB, down-regulated after HVST, but was not affected by LVLT. This indicated that WB promoted sphingolipid metabolism, whereas HVST inhibited its metabolism, which meant that HVST and WB exerted greater damage to sphingolipid metabolism than LVLT. As indispensable components of cell membranes, sphingolipid might be among the first cell component to encounter extracellular stresses (Liu et al., 2013). Fatty acids, especially unsaturated fatty acids, are known to induce decrease in cell respiratory activity, membrane fluidity, and coagulation of cytoplasmic materials, and eventually lead to cell lysis followed by leakage of macromolecules (Mousavi et al., 2016), and play important roles in the environmental stress. The cell membrane should retain its structural integrity as much as possible to antagonize the heat shock stress (Tian J. et al., 2017). Tian J. et al. (2017) reported that the decreased oleic acid content of *Saccharomyces cerevisiae* might be a self-protection mechanism of ethanol-adapted strains to maintain membrane integrity through decreasing membrane fluidity. In this study, the increased fatty acid content, especially unsaturated fatty acid might mean that the membrane integrity was damaged and the membrane fluidity of *E. coli* cells increased. The increase of lipid metabolites could mainly attribute to the heat of the treatments, which might result in remodeling the composition and structure of the cell membrane. This result was consistent with previous study that microorganisms could manifest increased resistance to environmental stress and control strategies after sublethal injury (Liu et al., 2018).

#### Amino Acid Metabolism

In general, stress could reduce membrane fluidity and accelerate the synthesis of some proteins (Zhang and Rock, 2008). The metabolomic analysis revealed that amino acid metabolism was strongly induced in *E. coli* cells by all the three treatments. There were 24, 24, and 20 differential compounds involved in amino acid metabolism after HVST, LVLT, and WB treatments, respectively. Among them, there were 15 amino acids overlapping the three treatments, and showed consistent trend of content change. However, in contrast to lipid-based metabolism, most of the differential amino acids were down-regulated, only indole and ketoleucine were up-regulated after LVLT. The observed increase in levels of indole and ketoleucine could attributed to protein denaturing and inhibition of protein synthesis, which might be caused by a halt in the synthesis of essential enzymes (Mousavi et al., 2016). Accordingly, the decreased levels of most amino acids might indicate a weakening in enzymatic activity related to protein degradation or a strengthening in enzymatic activity related to protein synthesis. For instance, glutamate is the direct ammonia assimilation product by glutamate dehydrogenase with high external ammonia concentrations, which subsequently serves as a primary precursor in multiple biosynthesis pathways, and it is usually synthesized more in actively growing cells (Lu et al., 2016). Glutamate was down-regulated after the three treatments, indicating that cell activity was reduced. The reductions of amino acids might reflect their consumption to produce new essential proteins or to repair damaged or misfolded proteins, in order to facilitate acclimation to the changing environment. The microorganisms are prone to rapidly changing when the environment conditions change, such as shifts in temperature, osmotic pressure, pH, or nutrient availability, and they have developed many strategies to cope with such unfavorable conditions. Among these strategies, acquisition of thermotolerance is mainly controlled by the activation and regulation of heat stress-related genes involved in the synthesis of specific compounds that protect the microorganism from thermal stress, which involves in induction of several proteins including stress proteins and chaperones (Paul et al., 2012; Dong et al., 2017). Therefore, in order to survive from the three treatments, *E. coli* cells might synthesize more molecular chaperones to regulate metabolism, which resulted in reduction of the most amino acids. Another reason for more down-regulation of amino acids might due to the ATP deficiency in response to heat shock from the three treatments, where synthesis of amino acids was required ATP as the energy source (Li et al., 2015).

#### Nucleotide Metabolism

As precursors of DNA and RNA, nucleotides participate in cell signaling and regulate many metabolic pathways, and play a vital role in stress response (Liu et al., 2013). Most purines and pyrimidines are present in the cell as nucleotides, and they are involved in the biosynthesis of genetic information carriers (DNA and RNA) or suppliers of energy (ATP and GTP) (Hu et al., 2017). The changes in purine and pyrimidine metabolism can suggest the increase of DNA damage and cell turnover (Zhou et al., 2017), where the nucleotide biosynthesis are direct indicators of DNA replication, cell division, and growth status, revealing a pronounced effect on cell proliferation (Bhat et al., 2015). The up-regulation and down-regulation changes of metabolites involved in nucleotide metabolism did not behave like lipid metabolism or amino acid metabolism, although more than half of the metabolites showed significant increase after the three treatments. Adenine was up-regulated after the three treatments, but its derivatives (adenosine, cAMP, ADP, and ATP) changed differently among the three treatments. Adenosine was down-regulated after HVST and WB, and was up-regulated after LVLT; cAMP was up-regulated after HVST and WB, and was down-regulated after LVLT; as one of the five kind nucleotides of DNA or RNA synthesis, the change of cAMP meant that the DNA or RNA synthesis was suppressed by HVST and WB, but was promoted by LVLT. In the meantime, ADP and ATP were only up-regulated after LVLT, the reason might be that the more energy was required in DNA synthesis during LVLT. Cytosine and its derivatives (5′-CMP and CTP) were all up-regulated after LVLT, this change was similar to adenines. Hypoxanthine was down-regulated after the three treatments, and the derivative inosine (IMP) could be converted to AMP and GMP, its up-regulation could promote generation of AMP and GMP during LVLT. In this study, guanine and its derivatives (cGMP and dGMP) were up-regulated after the three treatments, which meant that the IMP was mainly converted to AMP, and this result provided evidence that DNA or RNA synthesis were promoted. Uracil was down-regulated after HVST and WB, but was up-regulated after LVLT, which indicated that RNA synthesis was disturbed by the three treatments. However, UMP was down-regulated and UDP was up-regulated after LVLT, which meant that mRNA synthesis was promoted by LVLT; this result was consistent with the down-regulation of most amino acids, where some proteins were synthesized to resist stress. Previous study also proved that genes of *Streptococcus agalactiae* involved in purine metabolism were significantly up-regulated at 40°C than 30°C in the study of *S. agalactiae* transcriptomic analysis (Mereghetti et al., 2008).

#### Carbohydrate Metabolism

Several changes were observed in the levels of the metabolites that were involved in carbohydrate metabolism. Specifically, significant changes of metabolites (citric acid, malic acid, succinic acid, and lactic acid) related to tricarboxylic acid cycle (TCA cycle) were observed. Citric acid was up-regulated after HVST and LVLT, which was synthesized from oxaloacetic acid; but malic acid, as the precursor of oxaloacetic acid, was down-regulated after HVST and WB, and up-regulated after LVLT. Succinic acid was down-regulated after the three treatments, but lactic acid was only down-regulated after WB. The changes of metabolites involved in TCA cycle might be due to the energy requirement during synthesis of proteins and nucleotides. Additionally, citric acid is reported as a powerful chelator and it may play a role in managing concentrations of cations such as Ca^2+^ for survival, and there is evidence suggesting that Ca^2+^ is involved in the regulation of cell division and gene expression in response to external stimulation in prokaryotes (Alreshidi et al., 2015). Citric acid was up-regulated after HVST and LVLT, and the reason might be that the combination of citric acid and cations was damaged by the electric current during HVST and LVLT. α-ketoisovaleric acid, a branched-chain organic acid, served as a precursor in leucine and valine synthesis, the up-regulation after LVLT might be required by L-Valine synthesis (Li et al., 2017).

#### Energy Metabolism

Energy is required in basic metabolism, which includes syntheses of proteins, DNA, and RNA (Li et al., 2015). There were 8 metabolites involved in energy metabolism, 7 of them were presented after LVLT, but only 3 and 4 metabolites were presented after HVST and WB. The up-regulation of energy storage compounds (ATP and ADP) during LVLT indicated that they might be used to offset the negative effects of heat or the prolonged electric current stimulation from LVLT, thereby maintaining basic cellular reaction rates (Lu et al., 2016). Similar study reported that temperature variation perturbed the metabolic status of *S. agalactiae*, including energy metabolism processes, synthesis of proteins, contents of nucleotides, selective utilization of carbon sources, and some cellular materials (Hu et al., 2017). Another study on the metabolomic response of *E. coli* exposed to titanium dioxide nanoparticles also indicated that metabolites related to energy and growth were up-regulated (Planchon et al., 2017). The higher metabolomic changes of *E. coli* involved in energy metabolism might also be responsible for the increased heat tolerance of cells, also be partly responsible for the electric current from LVLT.

### Enrichment Analysis of the Differential Metabolites

**FIGURE 3:**
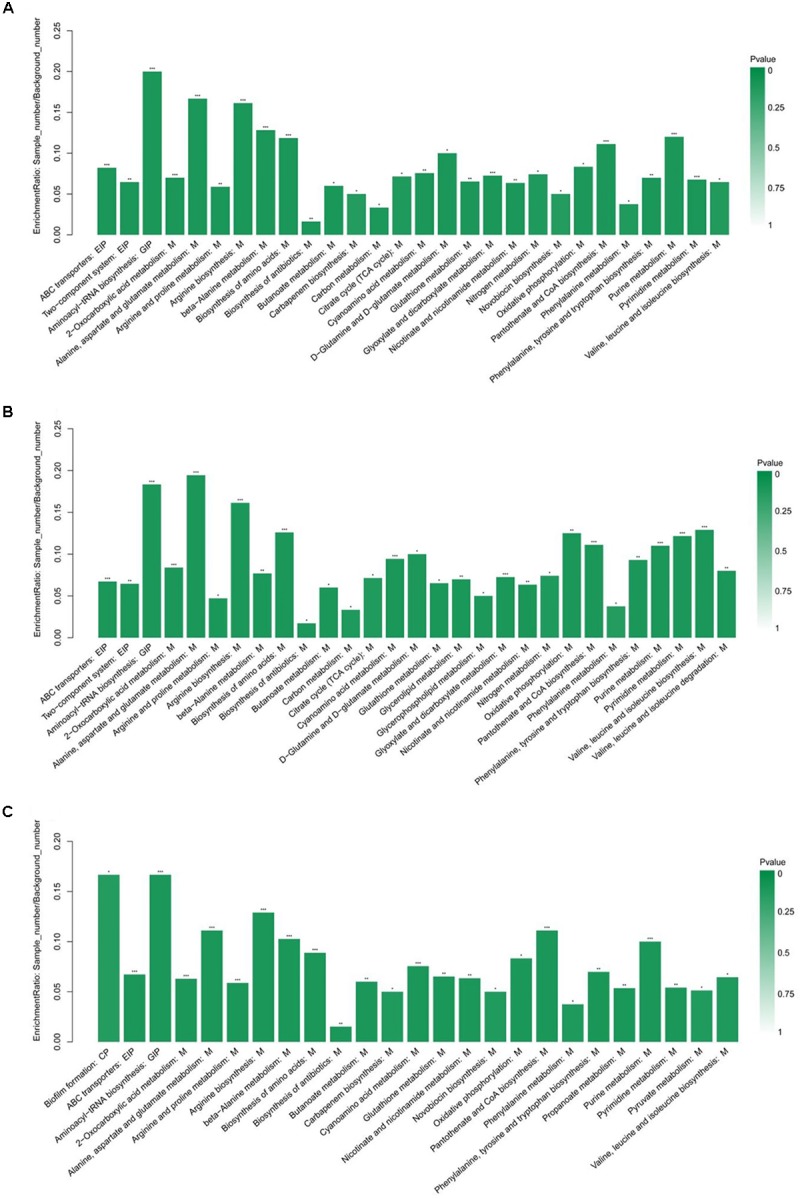
KEGG pathway enrichment analysis of differentially expressed metabolites from *E. coli* O157:H7 cells. **(A–C)** Showed the analysis for HVST vs. CT, LVLT vs. CT, and WB vs. CT treated samples, respectively. ^∗^ represented *P* < 0.05, ^∗∗^ represented *P* < 0.01, and ^∗∗∗^ represented *P* < 0.001.

The KEGG pathway enrichment analysis was performed by Fisher’s exact test, and those with *P* < 0.05 were considered significant pathways. This analysis could provide some additional clues about the complex identified metabolites. As shown in Figure 3, the statistical data revealed that differentially expressed metabolites were enriched to 28, 29, and 25 pathways from HVST vs. CT, LVLT vs. CT, and WB vs. CT treated *E. coli* cells, respectively. The top 5 pathways of enrichment ratio were aminoacyl-tRNA biosynthesis, 2-oxocarboxylic acid metabolism, alanine, aspartate and glutamate metabolism, arginine biosynthesis, beta-Alanine metabolism response to HVST; alanine, aspartate and glutamate metabolism, aminoacyl-tRNA biosynthesis, arginine biosynthesis, biosynthesis of amino acids, valine, leucine, and isoleucine biosynthesis response to LVLT; and aminoacyl-tRNA biosynthesis, biofilm formation, arginine biosynthesis, alanine, aspartate, and glutamate metabolism, and pantothenate and CoA biosynthesis response to WB, respectively. These results suggested that the most significantly changed metabolites mainly affect biosynthesis and metabolism of amino acid (alanine, arginine, aspartate, and glutamate, etc.) followed by aminoacyl-tRNA biosynthesis among the three treatments.

## Conclusion

In summary, in order to obtain similar inactivation levels of *E. coli* cells by HVST, LVLT, and WB, the required time for HVST (1.75 min) was shorter than LVLT (8.50 min) and WB (5.50 min). The major functional group of metabolites that displayed up-regulation after the three treatments were metabolites involved in lipid metabolism, while a down-regulation was metabolites involved in amino acid metabolism. On the whole, a stronger metabolomic response was caused by HVST and LVLT compared with WB, indicating that electric current might target partial metabolites during ohmic heating. This study provided a detailed description of overall metabolic responses of *E. coli* cells to ohmic heating, which would facilitate the understandings of ohmic heating on microbial inactivation on the molecular level. In addition, the results described here could provide a theoretical basis for ohmic heating on microbial inactivation in food products, and further facilitate the application of ohmic heating in food industry.

## Author Contributions

RD was the fund manager of the grants received from the National Key R&D Program of China (2016YFD040040302) and National Natural Science Foundation of China (No. 31271894), and directed and supervised the whole experimental and writing process. XT performed the research plan, experimental process, data analysis, and manuscript writing. QY participated in part of the experimental process and conducted part of the data analysis. DY and FJ participated in part of the experimental process. LS participated in part of the data analysis. XL participated in part of the research plan and provided valuable advice. ZL helped in the metabolomic analysis of microbes. TH helped in the draft revision of the manuscript.

## Conflict of Interest Statement

The authors declare that the research was conducted in the absence of any commercial or financial relationships that could be construed as a potential conflict of interest.

## Footnotes

1 http://www.hmdb.ca

2 https://metlin.scripps.edu
